# Hyperoxygenation Attenuated a Murine Model of Atopic Dermatitis through Raising Skin Level of ROS

**DOI:** 10.1371/journal.pone.0109297

**Published:** 2014-10-02

**Authors:** Hyung-Ran Kim, Jung-Hwan Kim, Eun-Jeong Choi, Yeo Kyong Lee, Jeong-Hae Kie, Myoung Ho Jang, Ju-Young Seoh

**Affiliations:** 1 Department of Microbiology, Ewha Womans University Graduate School of Medicine, Seoul, Republic of Korea; 2 Academy of Immunology and Microbiology (AIM), Institute for Basic Science (IBS), Pohang, Republic of Korea; 3 Division of Integrative Biosciences and Biotechnology (IBB), Pohang University of Science and Technology, Pohang, Republic of Korea; 4 Ewha Womans University High School, Seoul, Republic of Korea; 5 Pathology, National Health Insurance Cooperation Ilsan Hospital, Koyang, Republic of Korea; Clermont-Ferrand Univ., France

## Abstract

Atopic dermatitis (AD) is a chronic inflammatory skin disease resulting from excessive stimulation of immune cells. Traditionally, reactive oxygen species (ROS) have been implicated in the progression of inflammatory diseases, but several opposing observations suggest the protective role of ROS in inflammatory disease. Recently, we demonstrated ROS prevented imiquimod-induced psoriatic dermatitis through enhancing regulatory T cell function. Thus, we hypothesized AD might also be attenuated in elevated levels of ROS through tissue hyperoxygenation, such as by hyperbaric oxygen therapy (HBOT) or applying an oxygen-carrying chemical, perfluorodecalin (PFD). Elevated levels of ROS in the skin have been demonstrated directly by staining with dihydroethidum as well as indirectly by immunohistochemistry (IHC) for indoleamine 2,3-dioxygenase (IDO). A murine model of AD was developed by repeated application of a chemical irritant (1% 2,4-dinitrochlorobenzene) and house dust mite (*Dermatophagoide farinae*) extract on one ear of BALB/c mice. The results showed treatment with HBOT or PFD significantly attenuated AD, comparably with 0.1% prednicarbate without any signs of side effects, such as telangiectasia. The expressions of interleukin-17A and interferon-γ were also decreased in the AD lesions by treatment with HBOT or PFD. Enhanced expression of IDO and reduced level of hypoxia-inducible factor-1α, in association with increased frequency of FoxP3^+^ regulatory T cells in the AD lesions, might be involved in the underlying mechanism of oxygen therapy. Taken together, it was suggested that tissue hyperoxygenation, by HBOT or treatment with PFD, might attenuate AD through enhancing skin ROS level.

## Introduction

Atopic dermatitis (AD) is a common chronic inflammatory skin disease associated with substantial damage on the quality of life and economic burden [1]. AD results from dysregulated immune response due to excessive stimulation by external antigens. Currently, most cases of AD are treated by anti-inflammatory drugs such as topical corticosteroid or calcineurin inhibitors. While many patients of AD are managed tolerably, substantial number of patients still suffers from relapsing intolerable AD. Drug complications, such as telangiectasia or skin atrophy, still remain as problems [2].

Traditionally, reactive oxygen species (ROS) have been implicated in the progression of various kinds of inflammatory diseases, including AD. On the other hand, several studies reported association of enhanced autoimmunity with lowered levels of ROS. Autoimmune arthritis and experimental allergic encephalitis were aggravated in rodents with lower levels of ROS than wild type (WT) mice due to defects in ROS-producing enzyme system, such as neutrophil cytosolic factor (Ncf)-1 or nicotinamide adenine dinucleotide phosphate-oxidase (NOX)2 mutation [3]–[5]. In human, too, the risk of autoimmune diseases is increased in chronic granulomatous disease patients with lower levels of ROS than normal persons, due to defect in NOX [6]. To the contrary, elevated levels of ROS were reported to be associated with attenuated immunity. Allergen-induced airway inflammation and atherosclerotic lesions induced by high-fat diet were attenuated in mice with a higher level of ROS than WT mice due to the defect of a ROS metabolizing enzyme, glutathione peroxidase (*Gpx*)-1 [7], [8]. Mice with a higher level of ROS due to the defect of a non-enzymatic anti-oxidant, peroxiredoxin (Prx) II, were resistant to experimental colitis [9]. Recently, we also reported association of immune reactivity with ROS level both at lower and higher levels [10]. Imiquimod-induced psoriatic dermatitis was aggravated in *Ncf1* ^−/−^ mice, whereas attenuated in *Gpx1* ^−/−^ mice.

These experimental observations implied the immunoregulatory role of ROS [11]. In particular, the suppressive function of regulatory T cells (Tregs) seems to be closely linked to ROS level. *Ncf1* ^−/−^ Tregs were hypofunctional, while *Gpx1* ^−/−^ Tregs were hyperfunctional, compared with WT Tregs [10], [12]. Tregs were also hypofunctional in lowered levels of ROS prepared by adding antioxidants or NOX inhibitors, while hyperfunctional in elevated levels of ROS prepared by adding 2,3-dimethoxy-1,4-naphthoquinone (DMNQ) that increases intracellular ROS level. Differentiation of inducible (i) Tregs also seems closely linked to ROS level [13], [14]. Induction of FoxP3^+^ Tregs was attenuated, whereas that of T_H17_ cells was enhanced in lowered levels of ROS, and *vice versa* [3], [7], [9], [15], [16]. Taken together, ROS level is critical in the regulation of Treg function and differentiation. It is quite reasonable, as Tregs may contribute to the compensatory mechanism against the destructive effects that may be induced by ROS at high levels.

Several kinds of molecules may link ROS level and Treg function. Out of them, two immunoregulatory molecules were investigated in the present study. Indoleamine 2,3-dioxygenase (IDO) is induced by ROS and ROS enhance its enzyme activity, as superoxide radical acts as a cofactor [17]–[20]. IDO depletes tryptophan from the environment, starving effector cells, thus, results in immune suppression. Tryptophan depletion also results in expansion of Foxp3^+^ Tregs and inhibition of T_H17_ cell differentiation [21]–[23]. The second molecule is the transcription factor hypoxia-inducible factor (HIF)-1α. It was reported that, at hypoxic condition, HIF-1α promoted T_H17_ differentiation by directly inducing RORγt transcription and inhibited differentiation of Tregs by degrading FoxP3 protein [24]. Thus, IDO and HIF-1α may act as molecular cue that links oxygen and Treg, contributing to the preparation of immunosuppressive environment.

Hyperbaric oxygen therapy (HBOT) is a standard therapy for decompression sickness, gas embolism and CO poisoning [25], [26]. HBOT is also effective for gas gangrene, anaerobic infection, diabetic foot, Buerger’s disease and other oxygen-deficient conditions [27], [28]. Other than the current indications, many inflammatory or immune-mediated diseases were reported to be successfully treated by HBOT, implying immunomodulatory effects of HBOT [29]–[37]. Inflammatory and anti-inflammatory cytokines have been investigated in several studies, but the therapeutic mechanism of HBOT in immune-mediated diseases is not well-understood [31]. Recently, Faleo et al (2012) reported for the first time that FoxP3^+^ Tregs may be involved in the preventive mechanism of HBOT in autoimmune diabetes of NOD mice [38].

It has been well-known that HBOT increases cellular levels of oxygen and ROS [39], [40]. Therefore, HBOT may exert immunoregulatory effect through enhancing cellular level of ROS which is critical in the regulation of Treg function and differentiation. In the same way, other measures that can increase cellular ROS level might be as effective as HBOT for immune-mediated inflammatory diseases. Perfluorodecalin (PFD) has been developed and approved for clinical usage as a blood substitute due to its excellent oxygen-carrying capacity [41], [42]. PFD is also used in ophthalmic surgery to substitute for aqueous humor, as a contrast media for special MRI, and as a cosmetic ingredient for the purpose of oxygen delivery [43]–[45].

In this background, we hypothesized that augmented tissue oxygenation physically (HBOT) or chemically (PFD) might attenuate a murine model of AD. The results showed that treatment with HBOT or PFD attenuated AD comparably with corticosteroid, without any signs of side effects, such as telangiectasia.

## Materials and Methods

### Animals

Six week-old male BALB/c mice (23–27 g) were obtained from Daehan Biolink (Eumsung, Korea) and were maintained in specific pathogen-free conditions. This study was performed according to Korean Food and Drug Administration guidelines and was specifically approved by the Institutional Animal Care and Use Committee of Ewha Womans University Graduate School of Medicine (Permit Number: 10-0133).

### Experimental design

Mice were divided into 5 groups; the control group and AD groups which were further divided into 4 groups according to the treatment; AD – NT (not treated), AD – HBOT, AD – PFD, and AD – Steroid. Each group consisted of 12 mice which were accumulated from three separate experiments. Induction of AD using 2, 4-dinitrochlorobenzene (DNCB; Sigma, St Louis, IL) and *Dermatophagoide farinae* extract (DFE; Greer, Lenoir, NC) was performed as previously described (Fig. S1) [46]. Briefly, for the induction of AD, one of the two ear lobes was stripped five times with surgical tape (Nichiban, Tokyo, Japan), and 20 µl of DNCB (1%) was painted on each ear and then 20 µl of DFE (10 mg/ml) 3 days later. Alternate treatment of DNCB and DFE was repeated once a week for 4 weeks. For oxygen therapy, some mice were treated with HBOT or PFD before and during the induction of AD, as shown in Fig. S1. For comparison, other mice were treated with 0.1% prednicarbate cream (Green Cross, Yongin, South Korea). After six weeks, ear thickness was measured using a dial thickness gauge (Kori Seiki MFG, Co., Japan), blood was drawn to check serum IgE level, and the mice were sacrificed for histological examination.

### Hyperoxygenation

A hyperbaric oxygen chamber for animal study was purchased from Particla (Daejeon, South Korea). HBOT protocol is 100% O^2^ at 2.5 atm for 90 min after 10 min of compression, and then followed by 30 min of decompression. PFD (octadecafluorodecalin, 95% pure) was purchased from BOC Science (Shirley, NY). Twenty µl of PFD was applied onto the skin each time, which was absorbed well into the skin.

### Dihydroethidium staining

To measure *in* *situ* ROS level, frozen sections (10 µm) of ears were stained with 5 µM dihydroethidium (DHE, Molecular Probes Inc., Eugene, OR) in PBS for 30 min, rinsed, mounted, and observed using a fluorescent microscopy, according to the previously validated method [47], [48]. In the presence of O_2_^–^, DHE is converted to the fluorescent molecule ethidium, which can then label nuclei by intercalating with DNA.

### Histological examination

Excised ears were fixed in 4% paraformaldehyde for 16 h and embedded in paraffin. Thin (6 µm) sections were stained with hematoxylin and eosin (H & E) and were observed under a light microscope. Thickness of epidermis and area positive for fluorescence or immunohistochemical staining were measured by using Image J software (Image Processing and Analysis in Java, NIH, Bethesda, MD). For examination of mast cell infiltration, the sections were stained with toluidine blue and the number of mast cells was counted in five high-power fields (HPF) chosen at random in each slide by three different pathologists.

### IgE ELISA

The total concentration of IgE in the sera was measured by using Mouse IgE ELISA Quantitation Set purchased from Bethyl, Inc. (Montgomery, TX), according to the manufacturer’s instruction.

### Immuohistochemistry (IHC)

Sections were deparaffinated in xylene, dehydrated in ethanol and washed in PBS followed by successive permeabilization steps with 0.2% Triton in PBS. The sections were subjected to heat-induced antigen retrieval step before incubation with a universal blocking solution (Dako, Glostrup, Denmark) for 30 min. Then, the sections were incubated with anti-IL-17A (dilution fold 1/50, clone TC11-18H10, Novus Biologicals, Littleton, CO), anti-IFN-γ (dilution fold 1/100, goat polyclonal, R&D Systems, Minneapolis, MN), anti-IDO (dilution fold 1/50, rabbit polyclonal, Abcam, Cambridge, UK), anti-HIF-1α (dilution fold 1/40, rabbit polyclonal, Novus Biologicals) or anti-FoxP3 (dilution fold 1/100, rabbit polyclonal, Abcam) for 30 min at RT. For IL-17A, the sections were incubated with biotinylated rabbit anti-rat IgG antibody (Vector Laboratories, Burlingame, CA) and then developed using a streptavidin-horseradish peroxidase (HRP) complex (Vector Laboratories) and diaminobenzidine (DAB) substrate. For the others, the sections were incubated with biotinylated anti-rabbit, anti-mouse and anti-goat immunoglobulins followed by a streptavidin-HRP complex and DAB substrate organized in a commercial kit (Dako). The numbers of IL-17A^+^, IFN-γ^+^ or FoxP3^+^ cells were counted in five high-power fields (HPF) chosen at random in each slide by three different pathologists, and the IDO^+^ or HIF-1α^+^ area were measured in five fields chosen at random by using Image J software.

### Semi-quantitative reverse transcription-polymerase chain reaction (RT-PCR)

Total RNA was extracted from the ears of all the 12 mice in each group using a RNA extraction kit (Qiagen, Santa Clara, CA), according to the manufacturer’s protocol, and stored at −80°C until use. Then, cDNA was synthesized from 1 µg of each RNA sample using a cDNA synthesis kit (Enzynomics, Daejeon, Korea), and was amplified by PCR using the access RT-PCR system Takara thermal cycler (Takara, Seoul, Korea). The primer sets used in the PCR amplification were as follows: IL-17A (forward: 5′-TTTTCAGCAAGGAATGTGGA-3′, reverse: 5′-TTCATTGTGGAGGGCAGAC-3′); IFN-γ (forward: 5′-TCTGGAGGAACTGGCAAAAG-3′, reverse: 5′-TTCAAGACTTCAAAGAGTCTGAGG-3′); HIF-1α (forward: 5′-TCAAGTCAGCAACGTGGAAG-3′, reverse: 5′-TATCGAGGCTGTGTCGACTG-3′); FoxP3 (forward: 5′-CCAGAGAGAAGTGGTGCAGT-3′, reverse: 5′-GTGTCCTCTGCCTCTCCG-3′), and GAPDH (forward: 5′-GTCTTCTCCACCATGGAGAAGGCT-3′, reverse: 5′-CATGCCAGTGAGCTTCCCGTTCA-3′). The PCR products were separated on a 2% agarose gel containing ethidium bromide, visualized, and photographed using a gel documentation system (UVP, Cambridge, UK). Band intensity was quantified by densitometry using Image J software. The relative expression levels of target genes were normalized using GAPDH as an internal control.

### Statistics

Data are expressed as the mean ± SD (*n* = 12). Comparison of data was performed using Kruskal–Wallis one-way analysis of variance. *P* values less than 0.05 were considered statistically significant.

## Results

### Treatment with HBOT or PFD increased skin ROS level

**Figure 1:**
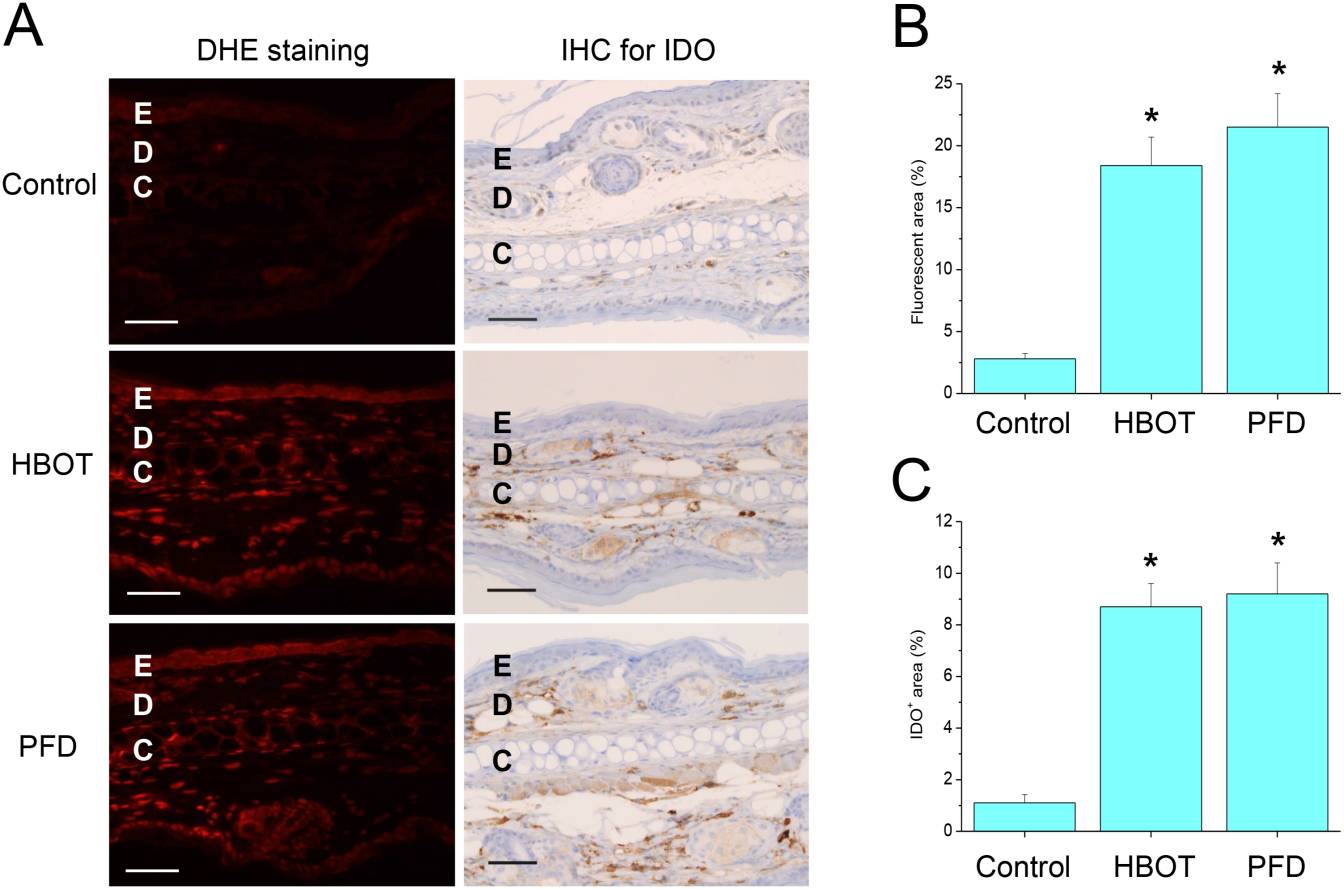
Treatment with HBOT or PFD increased skin ROS level. Fluorescent microscopic observation of the frozen sections stained with dihydroethidium (DHE), which reflects ROS level in the tissues (A, left). IHC of the paraffin sections for IDO, which reflects ROS level in the tissues indirectly (A, right). E, epidermal layer; D, dermal layer; C, cartilaginous layer. Scale bar is 100 µm. Fluorescent area (B) and IDO-expressing area (C) were measured by using image analysis software. Data are expressed as the mean ± SD (*n* = 12). *P<0.05, compared with the control.

Fluorescent microscopic examination of the ear of control mice stained with DHE showed little fluorescence without any visible nuclear fluorescence (Fig. 1A, Left). In contrast, fluorescence intensity was increased and fluorescent nuclei were clearly visible in the epidermal, dermal and cartilaginous layers in the ear of the mice treated with HBOT or PFD, suggesting increased level of ROS (Fig. 1B). IHC showed that IDO was very rarely observed in the dermal layer of the ear of normal mice (Fig. 1A, Right). In the mice treated with HBOT or PFD, IDO expression was increased both in terms of frequency and intensity, also implying that skin ROS level was increased (Fig. 1C). IDO-expressing cells were mostly observed in the dermal layers of the ears of the mice treated with HBOT or PFD.

### Treatment with HBOT or PFD attenuated AD

**Figure 2:**
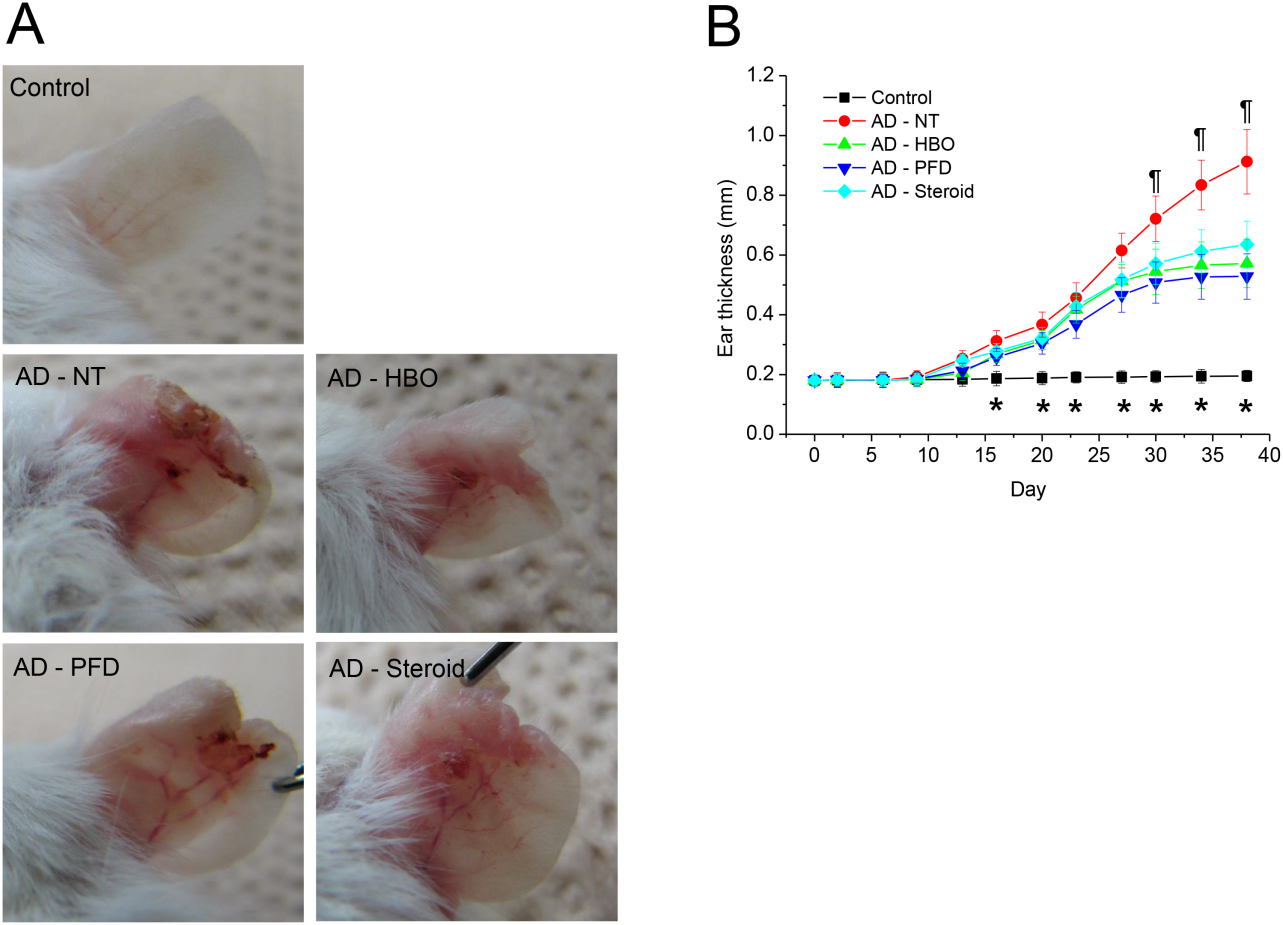
Treatment with HBOT or PFD decreased ear swelling in the AD lesions. Gross examination of the AD lesions at the end of the experiment (day 38) shows skin thickening, crust-formation and scaling, which was apparently attenuated by treatment with HBOT, PFD or steroid (A). Measurement of the ear thickness showed significant swelling in the AD lesions from day 16 (B). Ear swelling was significantly attenuated in the mice treated with HBOT, PFD or steroid from day 30. Data are expressed as the mean ± SD (*n* = 12). *P<0.05, compared between the control and other groups; ^¶^ *P*<0.05, compared between AD-NT and AD – HBO, AD – PFD or AD – Steroid groups.

**Figure 3:**
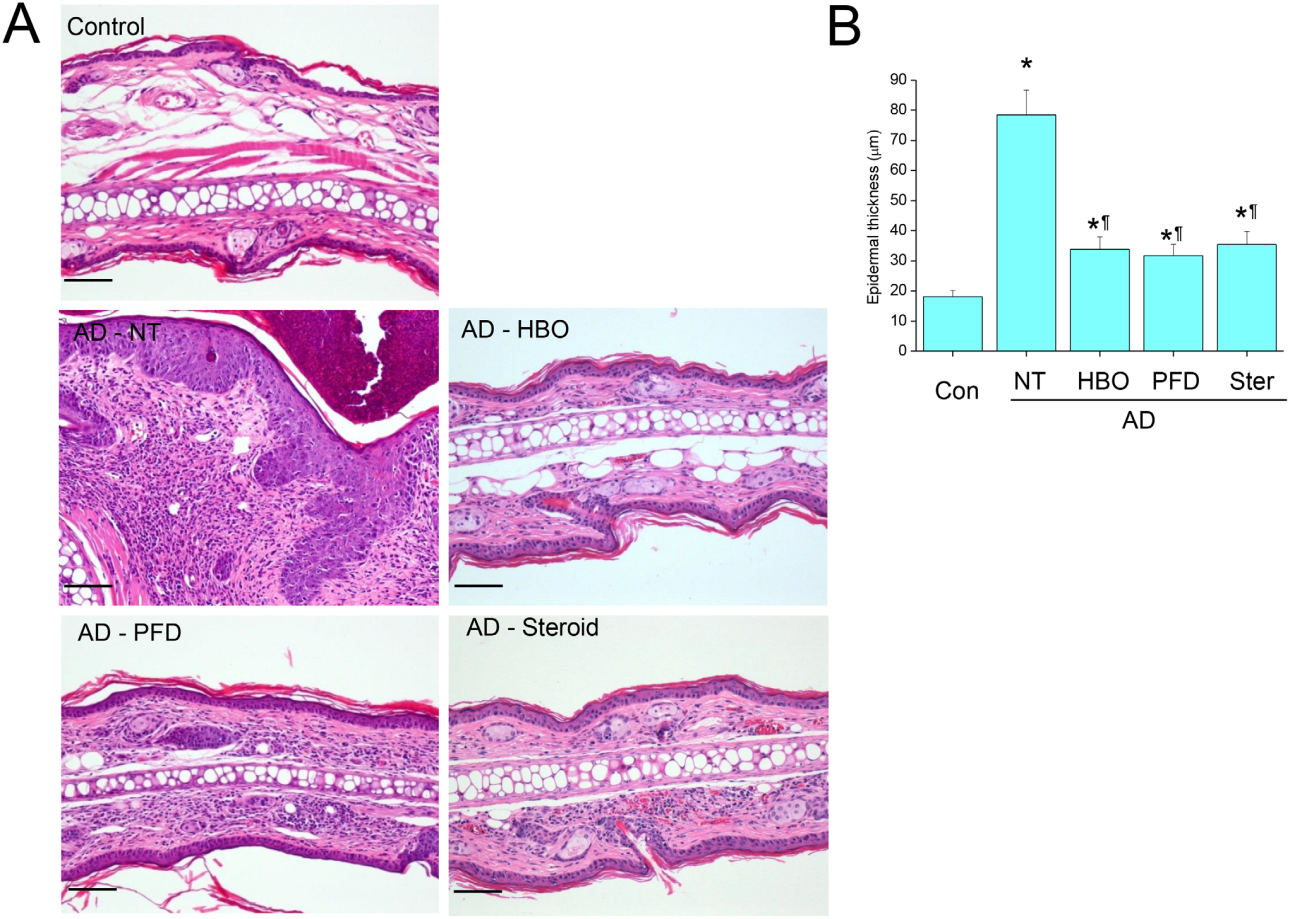
Treatment with HBOT or PFD attenuated inflammatory reaction in the AD lesions. Histological examination of the skin lesions of the AD model (A). H&E. Scale bar is 100 µm. Epidermal thickness measured by using image analysis software (B). NT, not treated. Data are expressed as the mean ± SD (*n* = 12). *P<0.05, compared with the control group; ^¶^ *P*<0.05, compared with the AD-NT group.

**Figure 4:**
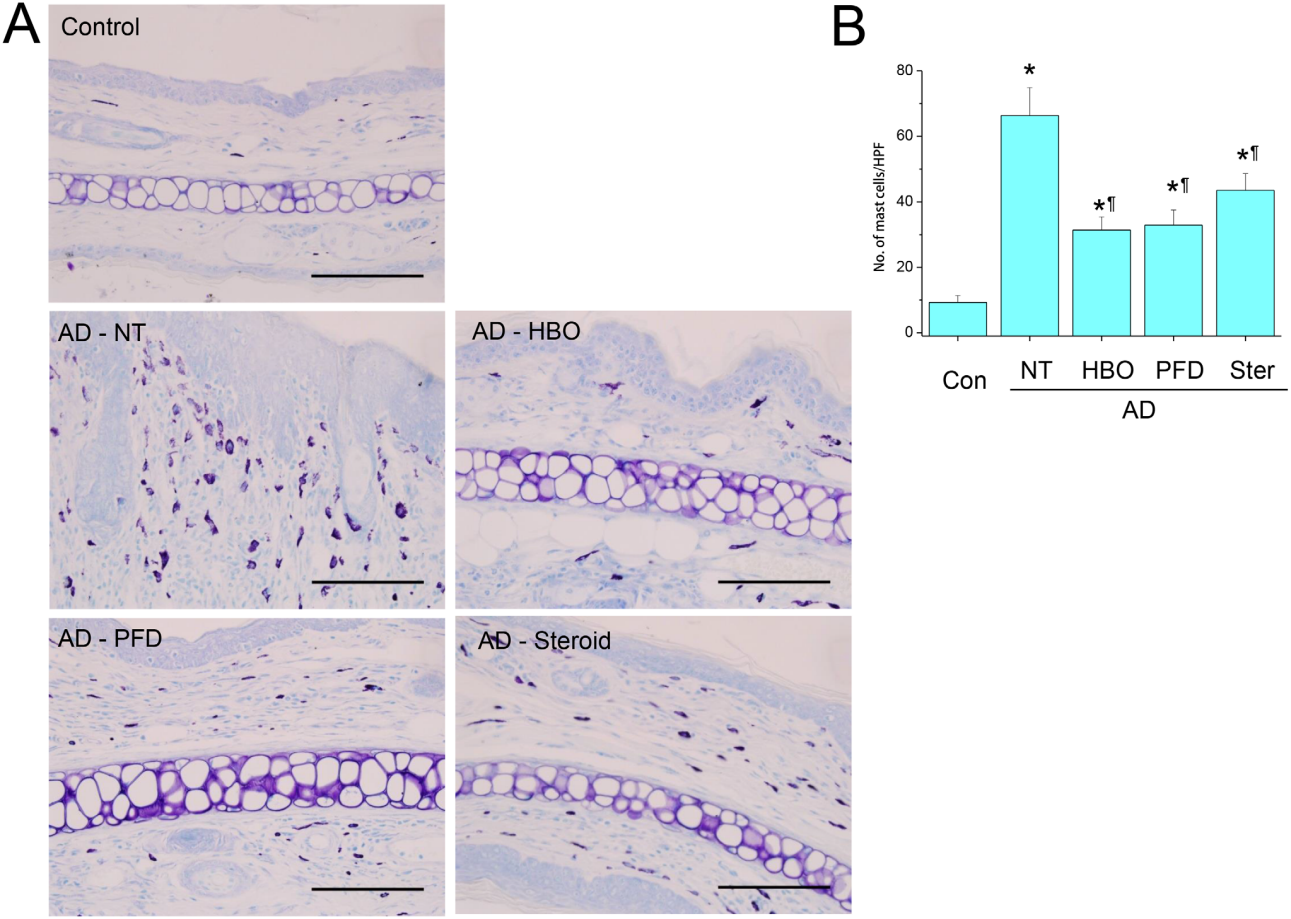
Treatment with HBOT or PFD decreased the frequency of mast cells in the AD lesions. Toluidine blue staining of the skin lesions of the AD model (A). Scale bar is 100 µm. The number of mast cells was counted in five high-power fields (HPF) chosen at random in each slide by three different pathologists (B). NT, not treated. Data are expressed as the mean ± SD (*n* = 12). *P<0.05, compared with the control group; ^¶^ *P*<0.05, compared with the AD-NT group.

**Figure 5:**
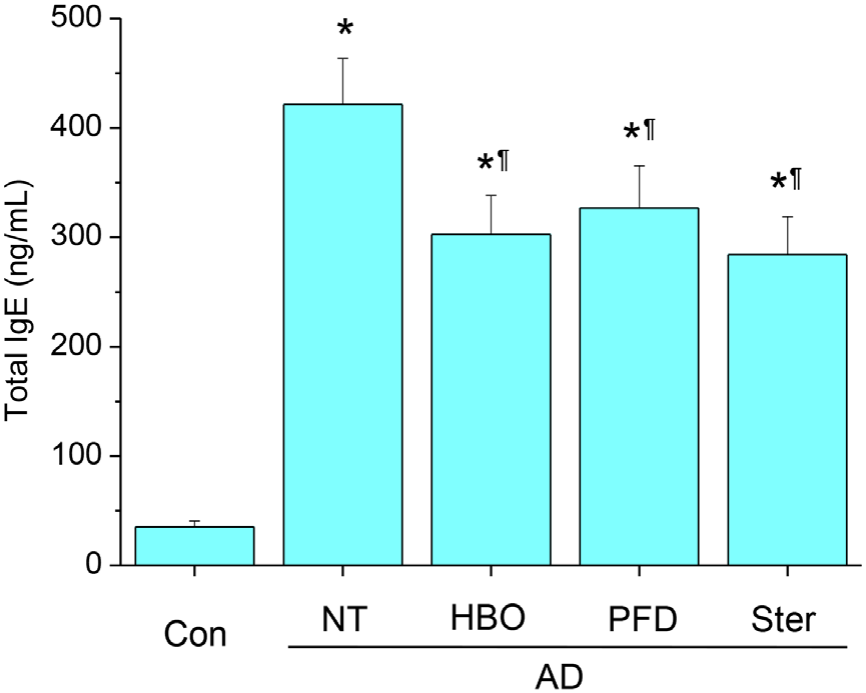
Treatment with HBOT or PFD decreased serum level of total IgE in the AD model mice. Serum levels of total IgE were measured by ELISA. NT, not treated. Data are expressed as the mean ± SD (*n* = 12). *P<0.05, compared with the control group; ^¶^ *P*<0.05, compared with the AD-NT group.

Mechanical stripping followed by repeated chemical irritation with DNCB and antigen challenge with DFE induced significant skin swelling on the ears (Fig. 2). Histological examination showed severe epithelial hyperplasia, exocytosis, hyperkeratosis, parakeratosis, perivascular inflammatory cell infiltration and dermal fibrosis (Fig. 3). Meanwhile, treatment with HBOT or PFD significantly decreased skin swelling in the AD lesions to the level comparable to those treated with steroid (Fig. 2 & 3). Histological examination of the AD lesions treated with HBOT or PFD showed only mild epithelial hyperplasia and perivascular inflammatory cell infiltration without any signs of telangiectasia (Fig. 3A). On the other hand, vascular dilation and microhemorrhage were frequently observed in the AD lesions treated with steroid, implying telangiectasia, although inflammatory reaction was also attenuated. Toluidine blue staining showed very rare mast cells in the control skin, while many mast cells were observed in the dermal layer of the AD lesions (Fig. 4A). The frequency of mast cells in AD lesions was also significantly decreased by treatment with HBOT or PFD to the level comparable to those treated with steroid (Fig. 4B). Compared with the control mice, serum level of total IgE was increased more than 10-fold in the AD model mice, which was slightly but significantly decreased by treatment with HBOT or PFD to the level comparable to those treated with steroid (Fig. 5).

### Treatment with HBOT or PFD decreased IL-17A and IFN-γ expression

**Figure 6:**
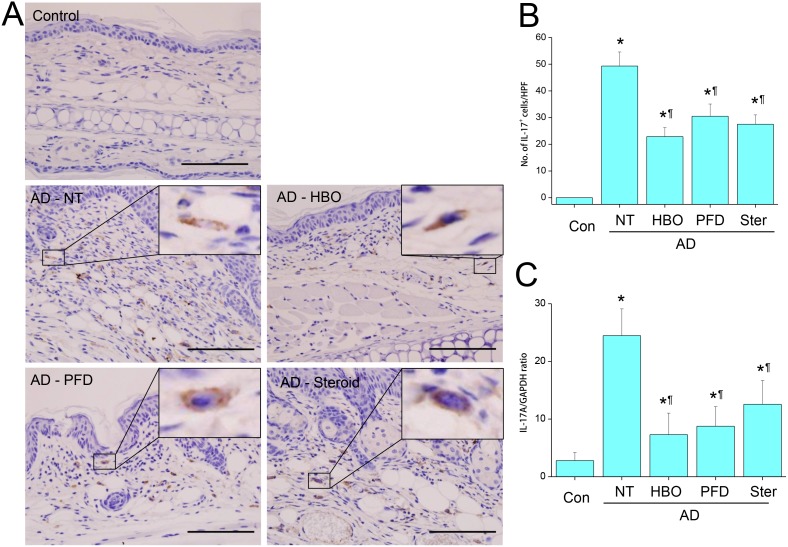
Treatment with HBOT or PFD decreased IL-17A expression in the AD lesions. IHC for IL-17A (A). Scale bar is 100 µm. The number of IL-17A-expressing cells was counted in five high-power fields (HPF) chosen at random in each slide by three different pathologists (B). RT-PCR for IL-17A (C). NT, not treated. Data are expressed as the mean ± SD (*n* = 12). *P<0.05, compared with the control group; ^¶^ *P*<0.05, compared with the AD-NT group.

**Figure 7:**
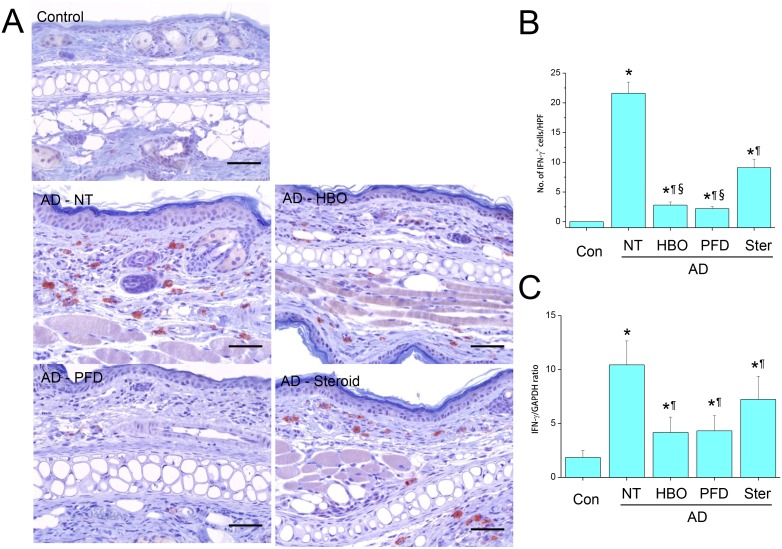
Treatment with HBOT or PFD decreased IFN-γ expression in the AD lesions. IHC for IFN-γ (A). Scale bar is 100 µm. The number of IFN-γ-expressing cells was counted in five high-power fields (HPF) chosen at random in each slide by three different pathologists (B). RT-PCR for IFN-γ (C). NT, not treated. Data are expressed as the mean ± SD (*n* = 12). *P<0.05, compared with the control group; ^¶^ *P*<0.05, compared with the AD-NT group; §, *P*<0.05, compared with the AD-Steroid group.

While IL-17A was not observed in the skins of the control mice, it was observed paly but obviously in the cytoplasm of infiltrating cells in the AD lesions by IHC (Fig. 6A). The frequency of IL-17A-expressing cells was significantly decreased by treatment with HBOT or PFD to the level comparable to those treated with steroid (Fig. 6B). IL-17A mRNA was induced in the lesions of AD, which was also significantly decreased by treatment with HBOT or PFD to the level comparable to those treated with steroid (Fig. 6C). IFN-γ was not observed in the control skins, but was observed vividly in the cytoplasm and surroundings of infiltrating cells in the AD lesions (Fig. 7A). IFN-γ-expressing cells were frequently observed in the dermal layers of the AD lesions. The frequency of IFN-γ-expressing cells was significantly decreased by treatment with HBOT or PFD (Fig. 7B). It was also decreased by steroid, but IFN-γ-expressing cells were still quite frequently observed in the dermal layers of the mice treated with steroid. IFN-γ mRNA was induced in the lesions of AD, which was also significantly decreased by treatment with HBOT or PFD (Fig. 7C). The level of IFN-γ mRNA in the lesions of AD treated with steroid was in between those treated with HBOT or PFD and those not treated.

### Treatment with HBOT or PFD enhanced IDO expression

**Figure 8:**
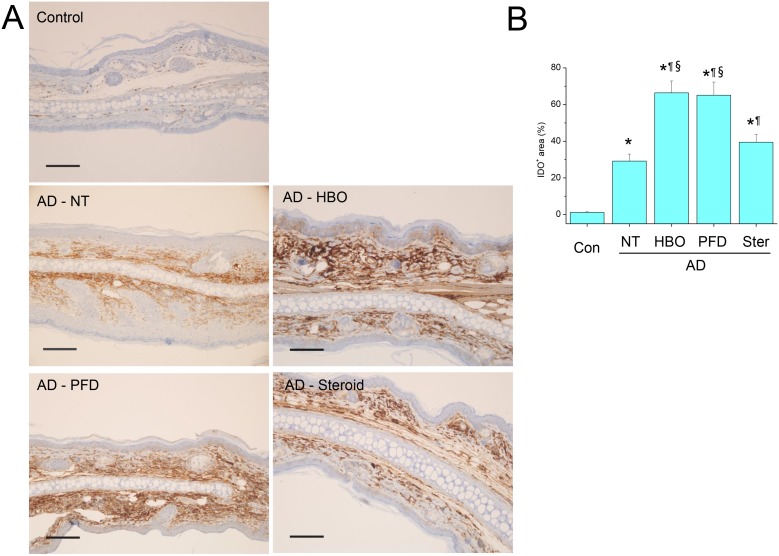
Treatment with HBOT or PFD enhanced IDO expression in the AD lesions. IHC for IDO (A). Scale bar is 100 µm. IDO^+^ area was measured in five fields chosen at random by using image analysis software (B). NT, not treated. Data are expressed as the mean ± SD (*n* = 12). *P<0.05, compared with the control group; ^¶^ *P*<0.05, compared with the AD-NT group; §, *P*<0.05, compared with the AD-Steroid group.

In the control skins, IDO was very rarely expressed in the dermal layers (Fig. 8A). In the AD lesions, IDO expression was markedly enhanced in many types of cells in the dermal layer, including infiltrating cells and fibroblasts. The proportion of IDO-expressing area in AD lesions was significantly increased by treatment with HBOT or PFD (Fig. 8B). In addition, the signal intensity of IDO was also stronger in the skins of the mice treated with HBOT or PFD than those not treated. Meanwhile, IDO expression in the AD lesions of the mice treated with steroid lied in between those treated with HBOT or PFD and those not treated.

### Treatment with HBOT or PFD decreased HIF-1α expression

**Figure 9:**
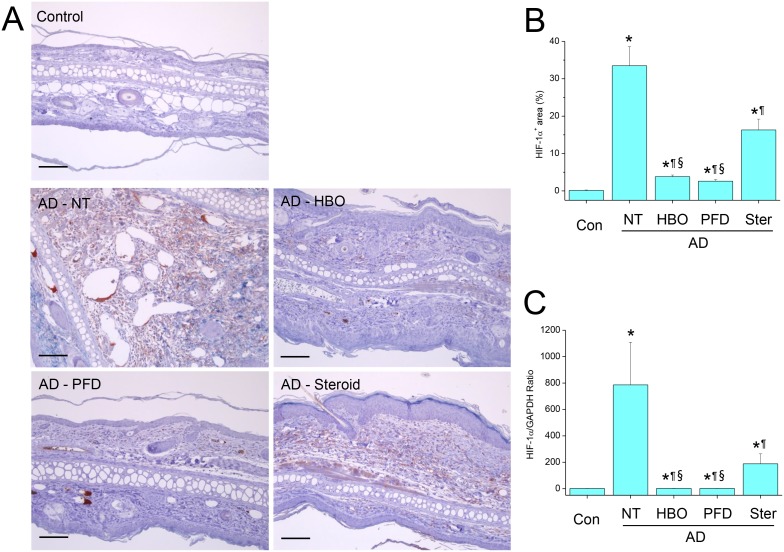
Treatment with HBOT or PFD decreased HIF-1α expression in the AD lesions. IHC for HIF-1α (A). Scale bar is 100 µm. HIF-1α^+^ area was measured in five fields chosen at random by using image analysis software (B). RT-PCR for HIF-1α (C). NT, not treated. Data are expressed as the mean ± SD (*n* = 12). *P<0.05, compared with the control group; ^¶^ *P*<0.05, compared with the AD-NT group; §, *P*<0.05, compared with the AD-Steroid group.

In the control skins, HIF-1α was not observed by IHC (Fig. 9A). On the other hand, HIF-1α was observed weakly but widespread in the dermal layer of the AD lesions, suggesting hypoxic condition in the inflammatory regions. In contrast, HIF-1α was hardly observed in the AD lesions treated with HBOT or PFD (Fig. 9B). Meanwhile, HIF-1α expression in the AD lesions treated with steroid lied in between those treated with HBOT or PFD and those not treated. HIF-1α mRNA was induced in the lesions of AD, which was also significantly decreased by treatment with HBOT or PFD (Fig. 9C). The level of HIF-1α mRNA in the lesions of AD treated with steroid was in between those treated with HBOT or PFD and those not treated.

### Treatment with HBOT or PFD increased the frequency of FoxP3^+^ Tregs

**Figure 10:**
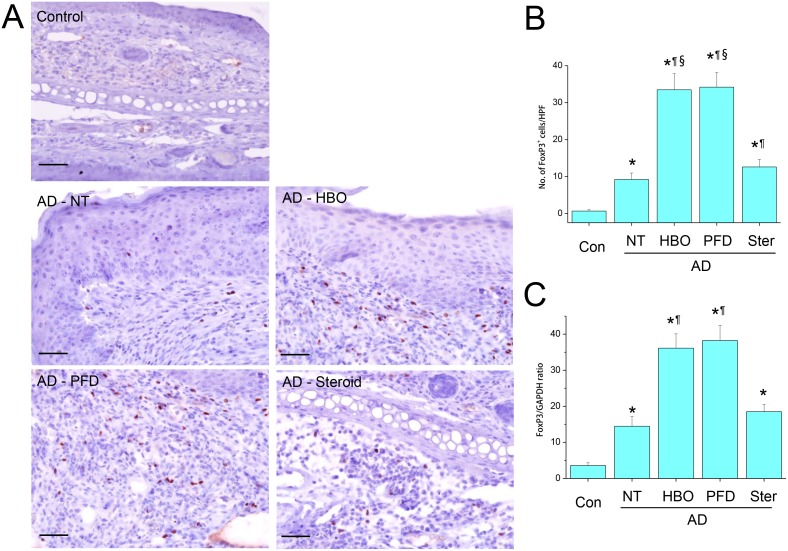
Treatment with HBOT or PFD increased the frequency of FoxP3^+^ Tregs in the AD lesions. IHC for FoxP3 (A). Scale bar is 100 µm. The number of FoxP3^+^ cells was counted in five high-power fields (HPF) chosen at random in each slide by three different pathologists (B). RT-PCR for FoxP3 (C). NT, not treated. Data are expressed as the mean ± SD (*n* = 12). *P<0.05, compared with the control group; ^¶^ *P*<0.05, compared with the AD-NT group; §, *P*<0.05, compared with the AD-Steroid group.

FoxP3^+^ cells were hardly observed in the control skins, but were observed in the AD lesions (Fig. 10A). The frequency of FoxP3^+^ cells was significantly higher in the AD lesions of the mice treated with HBOT or PFD than those not treated (Fig. 10B). The frequency of FoxP3^+^ cells in the AD lesions of the mice treated with steroid was in between those treated with HBOT or PFD and those not treated. FoxP3 mRNA was induced in the lesions of AD, which was significantly enhanced by treatment with HBOT of PFD (Fig. 10C).

## Discussion

In the present study, we demonstrated that augmented tissue oxygenation by treatment with HBOT or PFD attenuated a murine model of AD. Treatment with HBOT or PFD increased ROS level in the skin, thus elevated level of ROS might be involved in the immunoregulatory effect. This result is in accordance with others’ reports that elevated ROS level is closely associated with attenuation of inflammatory diseases [7]–[9]. We also reported recently that a murine model of inflammatory skin disease, imiquimod-induced psoriatic dermatitis, was attenuated in elevated level of ROS, whereas aggravated in lowered level of ROS [10].

These recent observations might be regarded to be contradictory to the traditional concept that ROS contribute to the progression of inflammatory diseases [49], [50]. Meanwhile, we proposed a new hypothesis that may compromise the traditional concept with the recent observations, considering the distinct fates of *Gpx1* KO mice and *Gpx1*×*Gpx2* double KO mice. *Gpx1* KO mice live as long as WT mice [51], [52]. In contrast, *Gpx1*×*Gpx2* double KO mice do not grow well and lifespan is shortened [53]. *Gpx1* KO mice are resistant to experimentally-induced inflammatory diseases [7], [8], whereas *Gpx1*×*Gpx2* double KO mice develop spontaneous inflammatory diseases, such as colitis [53]. Thus, the elevated level of ROS in *Gpx1* KO mice seems tolerable, whereas that in *Gpx1*×*Gpx2* double KO mice might be too high for the mice to tolerate. We can imagine a threshold level of ROS that divides intolerably higher levels and moderately elevated tolerable range. As ROS can induce direct tissue damage, defensive or compensatory mechanism counteracting the destructive effects should also be induced by ROS. Therefore, at moderately elevated levels of ROS below the threshold, compensatory mechanism may prepare anti-inflammatory environment while tissue damages are not yet induced by ROS. Attenuation of AD through tissue hyperoxygenation observed in the present study might be due to the anti-inflammatory environment prepared by moderately elevated levels of ROS through treatment with HBOT or PFD.

As we mentioned before, Tregs are hyperfunctional and iTreg differentiation is enhanced at moderately elevated levels of ROS, such as that in *Gpx1* KO mice [7], [10]. It is quite reasonable as Tregs play a key role in the maintenance of immune homeostasis *in* *vivo* [13], [14]. For the molecular mechanism that link ROS level and Treg function/differentiation, IDO and HIF-1α were investigated in the present study. IDO is primarily expressed in antigen-presenting cells, such as dendritic cells and macrophages, but IDO pathway is induced in many tissues during inflammation because IDO gene expression is induced by interferons [17], [19], [54]. Among them, IFN-γ is known to be the most powerful inducer of IDO. In the present study, IDO expression was significantly enhanced in the lesions of AD, compared with that in the control mice (Fig. 8). IDO expression was observed mainly in dermal layers in parallel with the distribution of IFN-γ-expressing cells. It is well-known that immune response pattern in AD lesion begins as T_H2_ type response, progresses to T_H17_ type response in acute stage and then reaches to T_H1_ type response in chronic stage [55]. In the present study, a weak expression of IFN-γ may be sufficient to induce IDO expression even before the chronic phase settles (Fig. 8). Meanwhile, in the lesions of the mice treated with HBOT or PFD, inflammatory reaction was very mild and IFN-γ-expressing cells were rarely observed. Albeit at minimal expression of IFN-γ, IDO expression was enhanced to an even higher level in the lesions of the mice treated with HBOT or PFD than in the AD lesions of the untreated mice (Fig. 8). The enhanced expression and stronger activity of IDO from the beginning due to elevated level of ROS by treatment with HBOT or PFD might contribute to the preparation of immunosuppressive environment preventing the development of inflammatory reactions in the AD lesions [21], [56]. In contrast, in the AD lesions of the untreated mice, IDO might be induced in later stage as a consequence of inflammatory reaction, thus contributing to the feedback regulation. In the lesions of the mice treated with steroid, IFN-γ-expressing cells were quite frequently observed although at a reduced frequency than that in the untreated mice. In parallel with the distribution of IFN-γ-expressing cells, IDO was also expressed at a high level in the AD lesions of the mice treated with steroid. Meanwhile, steroid has been also reported to increase ROS production in macrophages, thus can contribute to the induction and activation of IDO [57]. Actually, IDO expression was induced in the control skins by applying topical steroid (data not shown). However, presence of IFN-γ-expressing cells, although at a reduced frequency, suggests that the lesions of the mice treated with steroid have also gone into the chronic stage of AD. Accordingly, much of the IDO might be expressed as a consequence of T_H1_ type immune response in the lesions of the mice treated with steroid, while steroid itself might also induce IDO from the beginning by enhancing ROS production in macrophages. However, at the moment, we cannot dissect how much was expressed from the beginning and how much as the consequence of inflammatory response.

The second molecule investigated in the present study is a transcription factor HIF-1α. In response to hypoxia, HIF-1α mediates the metabolic switch from oxidative phosphorylation to anaerobic glycolysis [58]. Expression of HIF-1α is regulated at both post-translational and transcriptional levels [59]. At normoxia, the HIF-1α proteins are rapidly degraded by proteosome, resulting in essentially no detectable HIF-1α protein [60]. During hypoxia, HIF-1α becomes stabilized and translocates from the cytoplasm to the nucleus, where it dimerizes with HIF-1β, and the HIF complex becomes transcriptionally active. In addition to hypoxia, inflammatory signals can regulate HIF-1α activation. In the present study, HIF-1α protein was not detected in the control mice, but was detected in the AD lesions of the untreated mice by IHC, suggesting hypoxic condition in the inflammatory lesions (Fig. 9). However, it was not detected in the lesions of the mice treated with HBOT or PFD, suggesting that hypoxic condition was restored to normoxia in the lesions. Otherwise, attenuated inflammatory signals in the lesions of the mice treated with HBOT or PFD might not be sufficient for HIF-1α activation. At transcriptional level, HIF-1α mRNA was induced in the AD lesions, which was also suppressed by treatment with HBOT or PFD. As previously mentioned, HIF-1α activation favors T_H17_ response while suppressing Treg differentiation. Thus, reduced level of HIF-1α due to treatment with HBOT or PFD might also contribute to the preparation of immunosuppressive environment preventing the development of inflammatory reactions in AD lesions.

Enhanced expression of IDO and reduced level of HIF-1α in the AD lesions of the mice treated with HBOT or PFD might favor Treg-predominant immunosuppressive environment. Actually in the present study, FoxP^3+^ Tregs were observed at significantly higher frequencies in the lesions of the mice treated with HBOT or PFD (Fig. 10). This result is compatible with the report by Faleo et al (2012) [38]. However, the function of Tregs in the AD lesions could not be investigated due to the small number of cells and technical limit.

HBOT is a quite safe treatment modality that has been used for a long time in many clinical situations. Oxygen stress induced by HBOT is biochemically reversible, inducing no permanent changes [61]–[63]. PFD is also approved by FDA for the purpose of blood substitute, and is currently used as substitute for vitreous humor in ophthalmic surgery [43]–[45]. Therefore, we propose that tissue hyperoxygenation through HBOT or topical application of PFD might be an alternative therapeutic strategy for AD.

In the present study, we demonstrated the augmented tissue oxygenation by HBOT or PFD was effective in the prevention of AD. We also investigated the therapeutic effects of oxygen therapy for AD, by applying HBOT or PFD after establishment of AD. The results showed that ear swelling was not reduced, but inflammatory reaction was attenuated while fibrotic changes were still observed. Thus, we interpreted oxygen therapy might be also effective in the treatment of AD that had been already established by suppressing inflammatory reaction, but our experimental schedule was not long enough to observe the healing process by which the chronic fibrotic lesions became normalized.

In summary, we demonstrated that hyperoxygenation was associated with attenuation of a murine model of AD. We also showed that IDO expression was enhanced, but HIF-1α level was reduced, while the frequency of FoxP3^+^ Treg was increased in the AD lesions by hyperoxygenation. Taken together, immunosuppressive environment might be created by the oxygen-dependent molecules and cells, IDO, HIF-1α and Tregs, thus contributing to the attenuation of inflammatory reactions in AD lesions.

## Supporting Information

Figure S1 **Experimental schedule.**
(TIF)
