# Correction: Hyperoxygenation Attenuated a Murine Model of Atopic Dermatitis through Raising Skin Level of ROS

**DOI:** 10.1371/journal.pone.0361943

**Published:** 2026-11-03

**Authors:** The PLOS One Editors

## Notice of Republication

This article was republished on October 19, 2026 to correct the author list and remove Yeo Kyong Lee from the byline. Please download this article again to view the correct version. The originally published, uncorrected article and the republished, corrected article are provided here for reference.

## Supporting information

S1 File Originally published, uncorrected article. (PDF)

S2 File Republished, corrected article. (PDF)
