# Supplementary figures and images for: Hyperoxygenation Attenuated a Murine Model of Atopic Dermatitis through Raising Skin Level of ROS
Source: PLoS One. 2014 Oct 2;9(10):e109297. doi: 10.1371/journal.pone.0109297 (PMC4183587; doi:10.1371/journal.pone.0109297)

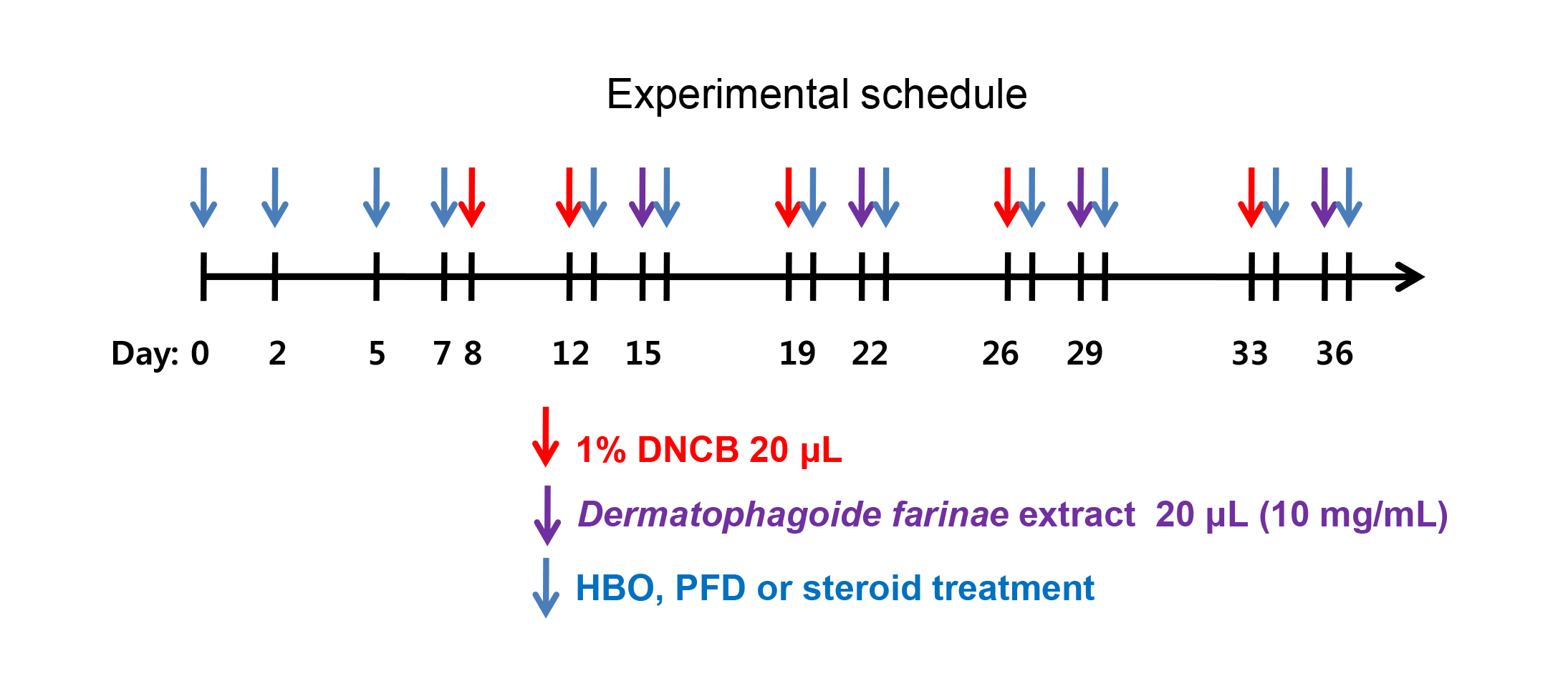

Supplement: Figure S1 — Experimental schedule. (TIF) [file pone.0109297.s001.tif]
